# Age-dependent predictors of soil multifunctionality in larch plantations: roles of nutrients and microbial networks

**DOI:** 10.3389/fpls.2026.1844613

**Published:** 2026-07-16

**Authors:** Yixuan Li, He Han, Zhaoxuan Ge, Zhidong Zhang

**Affiliations:** College of Forestry, Hebei Agricultural University, Baoding, China

**Keywords:** *Larix principis-rupprechtii*, microbial co-occurrence networks, microbial diversity, soil multifunctionality, soil nutrient dynamics, stand age

## Abstract

**Introduction:**

Understanding how stand development is linked to soil multifunctionality (SMF) is essential for sustaining ecosystem functions in plantation forests.

**Methods:**

In this study, five stand age gradients (21, 26, 32, 37, and 47 a) of larch (*Larix principis-rupprechtii*) plantations were analyzed in northern China. SMF was quantified using a multithreshold approach, and a structural equation model was employed to resolve pathways through which stand age influences SMF via soil physicochemical properties, microbial diversity, and co-occurrence network.

**Results:**

Soil organic carbon (SOC) and total nitrogen (TN) accumulated during early (21 and 26 a) to mid-aged stages (32 and 37 a). Specifically, SOC increased from 31.37 to 41.06 g·kg^−1^, while TN increased from 2.29 to 3.20 g·kg^−1^, before declining slightly in mature stands. Soil pH decreased from 6.67 to 6.31 along the stand age gradient, indicating intensified soil acidification. Microbial α-diversity increased from early to mid-aged stands and declined in mature (47 a) stands, with bacterial Chao1 index peaking at 37 a (*P* < 0.01) and fungal Chao1 index peaking at 32 a (*P* < 0.01). In contrast, microbial co-occurrence networks exhibited a continuous increase in structural complexity. SMF peaked in mature stands and was positively associated with SOC, TN, microbial α-diversity, and network complexity, but negatively associated with soil pH. Stand age influenced SMF primarily through indirect pathways. Specifically, SOC improved SMF mainly by promoting microbial α-diversity, whereas TN contributed primarily by strengthening bacterial network complexity. In contrast, soil pH exerted a persistent negative constraint on SMF by limiting microbial turnover. Notably, microbial network complexity explained variation in SMF more effectively than microbial diversity alone, particularly at higher multifunctionality thresholds.

**Discussion:**

Overall, SMF in larch plantations was linked to a hierarchical, multi-pathway cascade in which soil nutrient dynamics structured microbial interaction networks, thereby amplifying functional integration, and informing stand age–specific management strategies.

## 1 Introduction

Soil multifunctionality (SMF) describes the ability of soil ecosystems to concurrently support multiple ecosystem functions, including nutrient cycling, carbon sequestration, and biotic regulation (Su et al., 2021). As an integrative indicator of ecosystem functioning, SMF has become a central approach for assessing ecosystem stability, resilience, and service provision under environmental change (Lan et al., 2021). In forest ecosystems, soils constitute the fundamental biophysical interface linking vegetation dynamics, microbial processes, and biogeochemical cycles, thereby exerting a decisive influence on ecosystem productivity and long-term sustainability (Condit et al., 2013). Compared with assessments of single soil functions, the SMF framework captures functional synergies and trade-offs across multiple processes, offering a more holistic perspective on ecosystem functioning (Jing et al., 2015). Abiotic factors, including soil nutrients, pH and physical properties, are directly linked to soil biochemical processes and consequently correlate with multiple soil functions. In parallel, biotic factors, particularly microbial community composition, diversity, and interaction networks, play a key role in modulating SMF through their effects on nutrient transformation, organic matter decomposition, and soil biological regulation. Therefore, elucidating the biotic and abiotic predictors of SMF is critical for developing sustainable management strategies, particularly in planted forests facing degradation risks.

Soil microbial communities are central regulators of SMF due to their dominant roles in organic matter decomposition, nutrient mineralization, and energy transfer (Locey and Lennon, 2016). Bacteria and fungi, as the core components of the soil micro-food web, are linked to elemental cycling and plant–soil feedbacks through distinct yet complementary functional strategies (Jiao et al., 2021). Evidence suggests that bacterial diversity is more closely linked to SMF than fungal diversity, particularly in nutrient-limited systems (Guo et al., 2021; Liu et al., 2021; Wang et al., 2021). In contrast, fungi, especially saprotrophic and mycorrhizal groups, are widely recognized as key decomposers and sensitive indicators of soil ecological condition (Li et al., 2023; Li et al., 2025). Such functional differences between bacterial and fungal communities implies that taxonomic diversity alone is unlikely to fully explain variation in SMF. Instead, the identity of functionally important groups and their interaction structures may play a more decisive role. However, the relative contributions of microbial diversity versus microbial interaction structures to SMF remain insufficiently resolved, particularly in plantation forests undergoing stand development. Beyond microbial diversity per se, increasing evidence highlights the importance of microbial co-occurrence networks in sustaining ecosystem multifunctionality (Wagg et al., 2019; Kang et al., 2024). Co-occurrence networks characterize potential cooperative and competitive interactions among microbial taxa, thereby reflecting the structural organization and functional integration of microbial communities (Pržulj and Malod-Dognin, 2016). Complex networks often indicate a stable community capable of sustaining functions under environmental stress (Wagg et al., 2019; Guseva et al., 2022). Empirical studies have shown that microbial network complexity often explains SMF variation more effectively than species richness alone (Zhai et al., 2024), and that vegetation restoration can markedly enhance SMF through simultaneous increases in microbial diversity, β-diversity, and network complexity (Kang et al., 2025). Nevertheless, the specific contribution of network complexity versus diversity in regulating SMF remains poorly understood, particularly in ecosystems undergoing temporal succession.

Stand age represents a critical temporal gradient in plantation ecosystems that fundamentally alters soil habitats. As stands mature, shifts in vegetation-soil feedbacks modify soil physicochemical properties (e.g., pH, soil organic carbon). These abiotic shifts act as environmental filters, restructuring microbial community composition and their interaction networks (Ding and Eldridge, 2021; Hu et al., 2021; Li et al., 2025). For instance, soil acidification in aging coniferous stands may select for specific oligotrophic taxa or alter bacterial-fungal ratios, thereby cascading effects onto SMF. Despite this, the mechanistic pathways through which stand age influences SMF—specifically, whether it is primarily associated with abiotic resource accumulation, microbial diversity, or the topological restructuring of microbial networks—remain poorly understood.

Larch (*Larix principis-rupprechtii*), a dominant species in northern China, makes pivotal contributions to timber production, soil and water conservation, and regional ecological security (Cao et al., 2021). However, many larch plantations currently suffer from declining soil quality, reduced productivity, and weakened ecosystem stability as stands mature, constraining their capacity to deliver multifunctional ecosystem services (Cheng et al., 2023; Zhang et al., 2023). Currently, the understanding of the mechanisms through which stand age is associated with SMF though its influence on soil microbial diversity, co-occurrence network structure, and other factors remains inadequate.

To address these knowledge gaps, this study investigated a chronosequence of larch plantations in northern China, spanning five stand ages: 21, 26, 32, 37, and 47 years. We integrated analysis of soil physicochemical properties, microbial diversity, and co-occurrence network topology to identify the predictors of SMF. The specific objectives were: (1) to characterize the temporal trajectories of SMF, microbial community structure, and soil nutrients along the stand age gradient; (2) to explore the response of microbial network complexity to stand development; and (3) to disentangle the comparative roles of abiotic factors, microbial diversity, and network complexity in shaping SMF. We hypothesized that stand age is indirectly linked to SMF by modulating soil nutrient status and reshaping microbial network interactions, with network complexity acting as a key predictor of functionality.

## 2 Materials and methods

### 2.1 Study area

**Figure 1:**
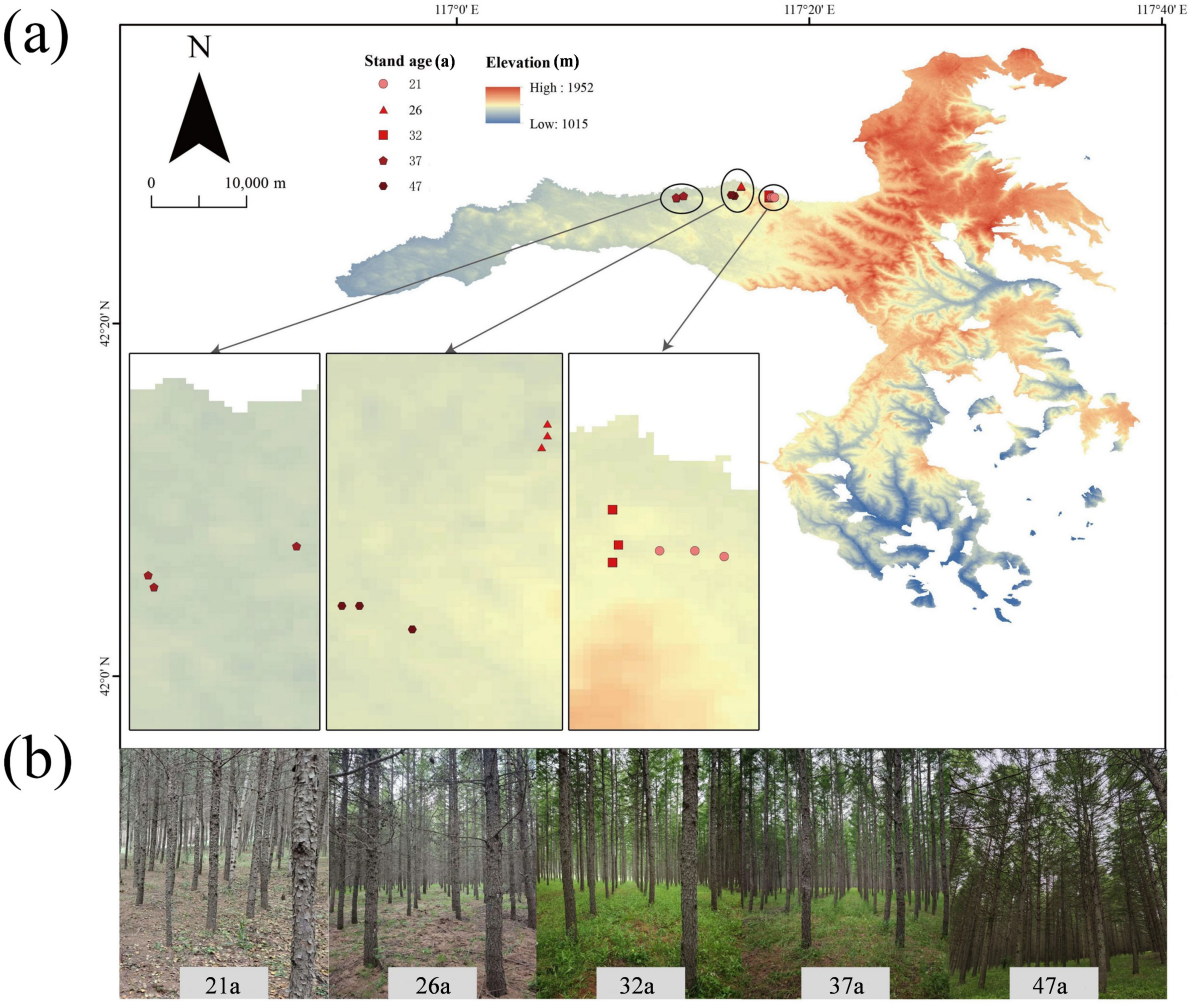
Location of the study sites in the Saihanba Mechanized Forest Farm, China. **(a)** The geographic distribution of larch plantation plots, and **(b)** pictures of stands with different developmental stages.

The fieldwork was performed at the Saihanba forest center (42°02′~42°36′N, 116°51′~117°39′E) in northern Hebei Province, China (**Figure 1**). This region represents a typical ecotone between the Greater Khingan Mountains and the Hunshandak Sandy Land, with elevations between 1010 and 1940 m. The region experiences a cold-temperate continental monsoon climate, with an average annual temperature of -1.3 °C and annual precipitation of 405–503 mm. The dominant soil types include mountain brown soils, aeolian sandy soils, and grey forest soils. Larch is the native and dominant afforestation species, covering approximately 76.2% of the plantation area (Zhang et al., 2022; Dai et al., 2025; Jia et al., 2025).

### 2.2 Experimental design and soil sampling

To minimize environmental heterogeneity, sampling plots were established in larch plantations characterized by comparable site conditions (soil type, slope, and aspect). Five representative stand ages (21, 26, 32, 37, and 47a) were selected. In this study, the 47-year-old stands were defined as mature stands because they represented the oldest stands in the sampling framework and approached the local rotation age (>50 years). The 21- and 26-year-old stands were classified as young to middle-aged stands, whereas the 32- and 37-year-old stands were categorized as near-mature stands. Stands were separated by 1–2 km to ensure independence. For each stand age, three 30 m × 30 m plots were randomly established at distances >50 m from forest edges and from each other, yielding a total of 15 plots (**Figure 1**). After litter removal, five random sampling points were established along diagonal transects in each plot. Soils from 0–10 cm and 10–20 cm were collected, sieved (0.25 mm), and composited by depth for each plot. These two soil layers were treated as distinct microhabitats rather than technical replicates, as they differ significantly in organic matter input, root density, and microbial biomass (see **Table 1**). The composite samples (30 samples in total) were classified into three subsamples: one stored at -80 °C for DNA extraction, one at 4 °C for multifunctionality assessments, and one air-dried for physicochemical analysis.

**Table 1:** Variations in soil physicochemical properties across stand ages and soil layers.

Standage/a	SWC/%		pH		SOC/(g·kg^-1^)		TN/(g·kg^-1^)		TP/(g·kg^-1^)		TK/(g·kg^-1^)		AN/(mg·kg^-1^)		AP/(mg·kg^-1^)		AK/(mg·kg^-1^)	
	0-10cm	10-20cm	0-10cm	10-20cm	0-10cm	10-20cm	0-10cm	10-20 cm	0-10cm	10-20cm	0-10cm	10-20cm	0-10cm	10-20cm	0-10cm	10-20cm	0-10cm	10-20cm
21	20.32 ± 0.83aA	16.56 ± 0.48aB	6.67 ± 0.06aA	6.87 ± 0.01aA	31.37 ± 1.12cA	11.40 ± 1.61dB	2.29 ± 0.05cA	1.73 ± 0.25dB	0.24 ± 0.10dA	0.14 ± 0.01dA	17.76 ± 0.02aA	17.19 ± 0.50aB	256.81 ± 3.10aA	146.91 ± 0.71cB	5.88 ± 0.57cA	2.56 ± 0.38cB	123.17 ± 4.65dA	117.65 ± 1.69cdA
26	17.75 ± 0.25bcA	15.11 ± 0.14bcB	6.60 ± 0.04bA	6.52 ± 0.03bA	36.10 ± 1.11bA	24.07 ± 5.71cB	2.57 ± 0.45bA	1.86 ± 0.14dB	0.29 ± 0.01cA	0.19 ± 0.02cB	17.83 ± 0.03aA	17.41 ± 1.33aA	269.68 ± 3.07aA	236.12 ± 1.54aB	6.78 ± 0.22cA	2.59 ± 0.39cB	132.47 ± 2.14cA	121.76 ± 0.33cB
32	16.99 ± 0.20cA	14.11 ± 1.11cB	6.49 ± 0.18cA	6.32 ± 0.03cA	35.64 ± 1.57bA	31.68 ± 2.17bA	2.77 ± 0.12bA	2.30 ± 0.15cA	0.40 ± 0.01bA	0.27 ± 0.01bB	18.07 ± 0.07aA	17.57 ± 0.13aB	189.22 ± 1.10bA	192.21 ± 7.81bA	8.88 ± 0.43bA	4.82 ± 0.40bB	218.63 ± 3.55bA	146.20 ± 0.49bB
37	18.60 ± 0.75bA	15.25 ± 0.27bB	6.41 ± 0.07cA	6.46 ± 0.02bcA	41.06 ± 0.33aA	36.52 ± 0.20aB	3.20 ± 0.17aA	2.83 ± 0.11aB	0.59 ± 0.02aA	0.30 ± 0.01abB	18.31 ± 0.13aA	17.45 ± 1.73aB	174.16 ± 3.84bA	120.03 ± 1.44dB	11.20 ± 1.85aA	7.27 ± 0.10aB	256.60 ± 1.98aA	180.48 ± 2.41aB
47	21.67 ± 0.49aA	17.10 ± 0.74aB	6.31 ± 0.05dA	6.48 ± 0.04bcA	41.47 ± 0.45aA	35.33 ± 0.32aB	3.18 ± 0.02aA	2.68 ± 0.05bB	0.43 ± 0.01bA	0.34 ± 0.02aB	17.51 ± 0.05aA	17.41 ± 0.25aA	163.76 ± 0.67bA	108.89 ± 1.39eB	10.14± 0.8aA	8.01 ± 0.31aA	256.57 ± 4.69aA	114.46 ± 1.04dB

SWC, soil water content; pH, soil acidity and alkalinity; SOC, soil organic carbon; TN, total nitrogen; TP, total phosphorus; TK, total potassium; AN, alkali-hydrolyzable nitrogen; AP, available phosphorus; AK, available potassium. Different lowercase letters indicate significant differences among stand ages (*P* < 0.05); different uppercase letters indicate significant differences between soil layers *(P* < 0.05).

### 2.3 Soil physicochemical analyses

Soil water content (SWC) was determined gravimetrically by drying at 105 °C for 72 h. Soil pH was measured with a calibrated pH meter. Soil organic carbon (SOC) was quantified using the potassium dichromate oxidation method. Total nitrogen (TN) was quantified using the semi-micro Kjeldahl method. Total phosphorus (TP) by molybdenum blue colorimetric, and total potassium (TK) by flame photometry. Available nutrients were extracted and measured as follows: available phosphorus (AP) using sodium bicarbonate extraction-colorimetry; available potassium (AK) using flame photometry; and alkali-hydrolyzable nitrogen (AN) via the alkali hydrolysis diffusion method (Bao, 2000).

### 2.4 DNA extraction and amplification sequencing

Genomic DNA extraction was performed using the TGuide S96 Kit (Tiangen Biotech Co., Ltd., Beijing, China). DNA quality was determined with a Synergy HTX microplate reader (GeneCompang Limited), and only samples meeting sequencing quality requirements were retained for subsequent analyses. PCR amplification was performed on the target gene fragments, and successful amplification was verified by electrophoretic separation on 1.8% agarose gels. The V3-V4 region of the bacterial 16S rRNA gene was selected for amplification with the primer pair 338F (5’-ACTCCTACGGGAGGCAGCAG-3’) and 806R (5’-GGACTACHVGGGTWTCTAAT-3’) to assess bacterial community composition. Fungal community composition was assessed by amplifying the ITS1 region with the primer set ITS1F (5’-CTTGGTCATTTAGAGGAAGTAA-3’) and ITS2R (5’-GCTGCGTTCTTCATCGATGC-3’). PCR amplicons were then purified, quantified, and normalized to construct sequencing libraries, the quality of which was evaluated using a Qsep-400 system. Following quality assessment, qualified libraries were processed for paired-end sequencing on an Illumina NovaSeq 6000 system. Raw sequencing reads were first quality-filtered using Trimmomatic v0.33, after which primer sequences were identified and trimmed using Cutadapt v1.9.1 to yield high-quality clean reads. All downstream bioinformatic processing was conducted in the QIIME2 platform. Using the DADA2 plugin, sequence quality filtering and denoising were conducted, followed by paired-end read assembly and chimera filtering, resulting in a curated dataset of effective non-chimeric sequences (Liu et al., 2024). The raw sequencing depth of each sample is provided in **Supplementary Table 3**, and the rarefaction depth used for downstream analyses was 4732 reads.

### 2.5 Construction and analysis of microbial co-occurrence networks

Microbial co-occurrence networks were constructed separately based on ASV relative abundance data across all samples. To ensure robustness, only ASVs with an average relative abundance >0.1% was retained. To address the compositional nature of the data, the ASV table was normalized using cumulative sum scaling (CSS), and SparCC correlation matrices were calculated, and significant associations were identified using permutation-based null models. Correlations with |R| > 0.6 and FDR-corrected *P* < 0.05 were retained, where positive and negative correlations were interpreted as potential synergistic and competitive interactions, respectively. Network topology was characterized using key metrics including node number, edge number, average clustering coefficient, network diameter, modularity, graph density, and average path length. Network complexity, quantified as link density, was used as an integrative indicator of network connectivity (Luo et al., 2025). To represent overall network structure, three metrics (complexity, centrality and network diameter) were integrated into a single composite index by averaging their standardized values. This composite index was used as bacterial network complexity (BNC) and fungal network complexity (FNC) in subsequent regression analyses and structural equation modeling (SEM). Subnetwork matrices were extracted at the sample level using the *igraph* package in R software (Wagg et al., 2019; Jiao et al., 2022), and networks were visualized using Gephi software.

### 2.6 Microbial community composition analysis

**Figure 2:**
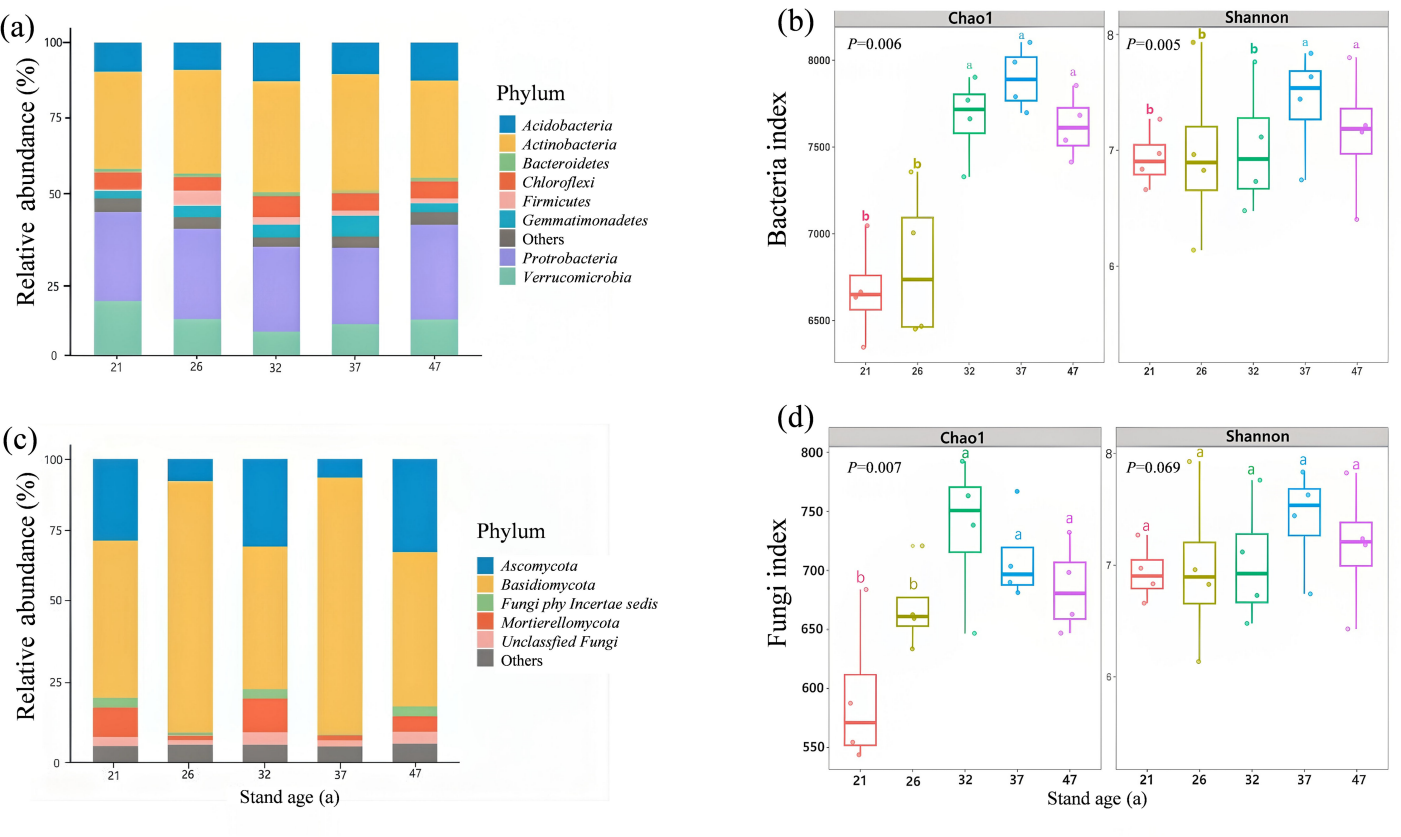
Stand age–dependent patterns of soil microbial diversity and community composition. **(a)** Mean relative abundances of dominant bacterial phyla along the stand-age gradient. **(b)** Variation of bacterial a-diversity (Chao1 and Shannon indices) among stand ages. **(c)** Mean relative abundances of dominant fungal phyla across the stand-age gradient. **(d)** Variation of fungal a-diversity (Chao1 and Shannon indices) among stand ages.

Principal coordinate analysis (PCoA) based on the Bray–Curtis dissimilarity matrix was performed to visualize differences in bacterial and fungal community composition among stand ages. Permutational multivariate analysis of variance (PERMANOVA, Adonis) with 999 permutations was applied to test the statistical significance of community structural differences, and the corresponding p-values were presented in **Figure 2**. Microbial β-diversity was further quantified using Bray–Curtis dissimilarity matrix. Mantel test was used to examine the correlation between bacterial and fungal β-diversity and SMF.

### 2.7 Quantification of soil multifunctionality

A total of 19 functional indicators representing soil nutrient status, microbial diversity, and network structures was initially considered (Vandandorj et al., 2017; Eldridge and Delgado-Baquerizo, 2018; Eldridge et al., 2020). To reduce multicollinearity, principal component analysis (PCA) was used to select representative variables, including SOC, pH, TN, microbial α- and β-diversity indices, and microbial network complexity (**Supplementary Tables 1**, **S2**).

Several approaches, including the single-function, turnover, averaging, and threshold methods, were used to assess SMF (Lan et al., 2023). However, the single-function approach fails to capture synergistic interactions among multiple soil processes (Wagg et al., 2014), whereas the turnover method does not directly reflect the overall magnitude of SMF (Chen et al., 2020). Similarly, the averaging method tends to obscure intrinsic correlations and trade-offs among individual functions, potentially leading to biased SMF estimates (Maestre et al., 2012). Given these limitations, the multi-threshold approach was adopted to quantify SMF, as this method has been widely validated and recommended in ecological studies for providing a comprehensive and threshold-sensitive assessment of SMF.

Each functional indicator was standardized relative to its maximum observed value, and SMF was calculated as the number of functions exceeding predefined thresholds (Byrnes et al., 2014; Manning et al., 2018). The multi-threshold method was implemented using the ‘getFuncsMaxed’ function in the *multifunc* R package. Thresholds of 25%, 50%, 75%, and 90% (Zirbel et al., 2019) were used to capture variation in SMF across stand ages. The SMF index was calculated as follows (Equation 1):

(1) $$SMF=\sum_{i=1}^{n}\left(r_{i}(f_{i})>t_{i}\right)$$

where *n* is the number of soil functional indicators, *f*_i_ is the measured value of the ith indicator, *r_i_* is a transformation function used to ensure positive directionality, and *t*_i_ is the applied threshold.

Our approach to constructing the SMF index, which incorporates both soil physicochemical properties (SOC, TN, pH) and microbial attributes (diversity, network complexity), follows established frameworks widely adopted in the SMF literatures (Delgado-Baquerizo et al., 2016; Luo et al., 2025). Following these precedents, SMF was conceptualized in the present study as an integrative indicator reflecting the overall soil biogeochemical capacity. In subsequent analyses, some variables included in the SMF index were further evaluated as explanatory variables to test hypothesized ecological pathways, thereby serving distinct analytical functions at different hierarchical levels. Given the inherent mathematical interdependence among these variables, the associations identified in this study should be interpreted as descriptive patterns of covariation rather than strictly causal relationships.

### 2.8 Statistical analysis

In all statistical analyses, statistical significance was determined at *P* < 0.05. However, for the analyses of drivers of SMF across multiple thresholds, a more stringent significance threshold (*P* < 0.001) was used to minimize the likelihood of false-positive results arising from multiple comparisons. Differences in soil physicochemical properties among stand ages and soil depths were tested using one-way ANOVA, followed by Tukey’s HSD *post hoc* test. Principal coordinate analysis (PCoA) was employed to assess age-related shifts in microbial community structure. Relationships between SMF and its potential predictors were evaluated using linear regression across SMF thresholds. Structural equation modeling (SEM) was performed using the *lavaan* package (Rosseel, 2012) to quantify direct and indirect effects of biotic and abiotic factors on SMF across all four threshold levels. In the SEM, bacterial and fungal α-diversity were represented by a composite index calculated as the mean of the standardized Shannon and Chao1 indices. To minimize multicollinearity, variance inflation factors (VIFs) were calculated for all predictor variables using the *vif* function in the *car* package. During model optimization, non-significant paths with standardized coefficients< 0.1 were iteratively removed (García‐Palacios et al., 2015). Model fit was evaluated using χ² tests and corrected Akaike Information Criterion (AIC_C_). All analyses were performed in R (version 4.5.2).

## 3 Results

### 3.1 Variations in soil physicochemical properties

Stand age and soil depth significantly influenced soil physicochemical properties (**Table 1**). For the majority of variables, values were significantly greater in the 0–10 cm soil layer compared with the 10–20 cm layer (*P* < 0.05), with more obvious vertical variation observed for SOC, AN, and AP. In the 0–10 cm layer, SWC was relatively higher in the 21a and 47a stands (*P* < 0.05), showing a decline followed by recovery with stand age. Soil pH decreased monotonically along the chronosequence and reached its lowest value in the 47a stand. Soil nutrients, including SOC, TN, AP, AK, and TP, peaked in the 37a stand. In contrast, AN was highest in the 26a stand and declined thereafter, whereas TK did not differ significantly among stand ages. In the 10–20 cm layer, SOC, TN, and AK exhibited trends similar to those in surface soil, while TP and AP increased significantly across the stand age sequence. AN declined after the 26a stand, and TK decreased in the 37a stand before partially recovering in the 47a stand.

### 3.2 Variations in microbial diversity and composition

Bacterial communities were dominated at the phylum level by *Actinobacteria*, *Proteobacteria*, and *Acidobacteria*, showing minimal shifts in relative abundance across the age gradients (**Figure 2b**). Fungal communities were dominated by *Basidiomycota* and *Ascomycota* (**Figure 2d**). These two dominant phyla exhibited an inverse relationship; notably, *Basidiomycota* exhibited variable relative abundance, with higher proportions observed in the 26- and 37-year-old stands, whereas *Ascomycota* predominated in the 21-, 32-, and 47-year-old stands. Soil microbial α-diversity exhibited clear stand age–dependent patterns (**Figure 2**). For bacterial communities, both Chao1 and Shannon variables showed significant increases along the stand age gradient (Chao1: *P* < 0.01; Shannon: *P* < 0.01; **Figure 2a**). The lowest bacterial α-diversity was observed in the early stages (21 and 26a), followed by a marked increase in mid-aged stages (32 and 37a), after which diversity tended to stabilize or slightly decline in the mature stage (47a). Fungal communities displayed a similar but less pronounced response to stand age (**Figure 2c**). Fungal richness (Chao1) showed significant increases along the stand age gradient (*P* < 0.01), whereas Shannon diversity showed only a marginal stand age effect (*P >*0.05). Fungal Chao1 values were lowest in the early stages and peaked in the 32a stands, followed by a slight decrease in 37a and 47a stands.

**Figure 3:**
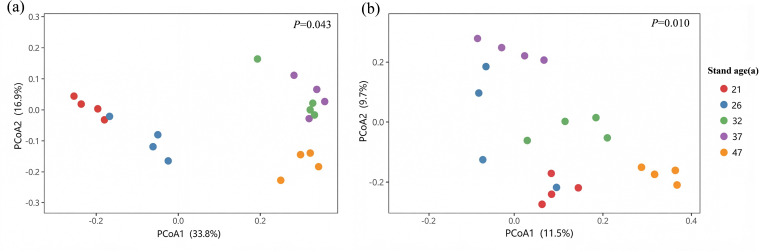
Principal coordinate analysis (PCoA) of community composition and structure for **(a)** soil bacterial and **(b)** fungal communities in Larix principisrupprechtii plantations across the stand-age gradient.

PCoA revealed significant compositional differentiation in both bacterial (*P* < 0.05) and fungal (*P* < 0.01) communities across stand ages (**Figure 3**). Bacterial communities showed a significant separation among stand ages, which explained 33.8% (PCoA1) and 11.5% (PCoA2) of the total variation. Fungal communities exhibited an even stronger differentiation among stand ages, with PCoA1 and PCoA2 accounting for 11.9% and 13.7% of the variation, respectively. The results indicated a progressive restructuring of bacterial and fungal assemblages with stand development.

### 3.3 Microbial co-occurrence networks

**Figure 4:**
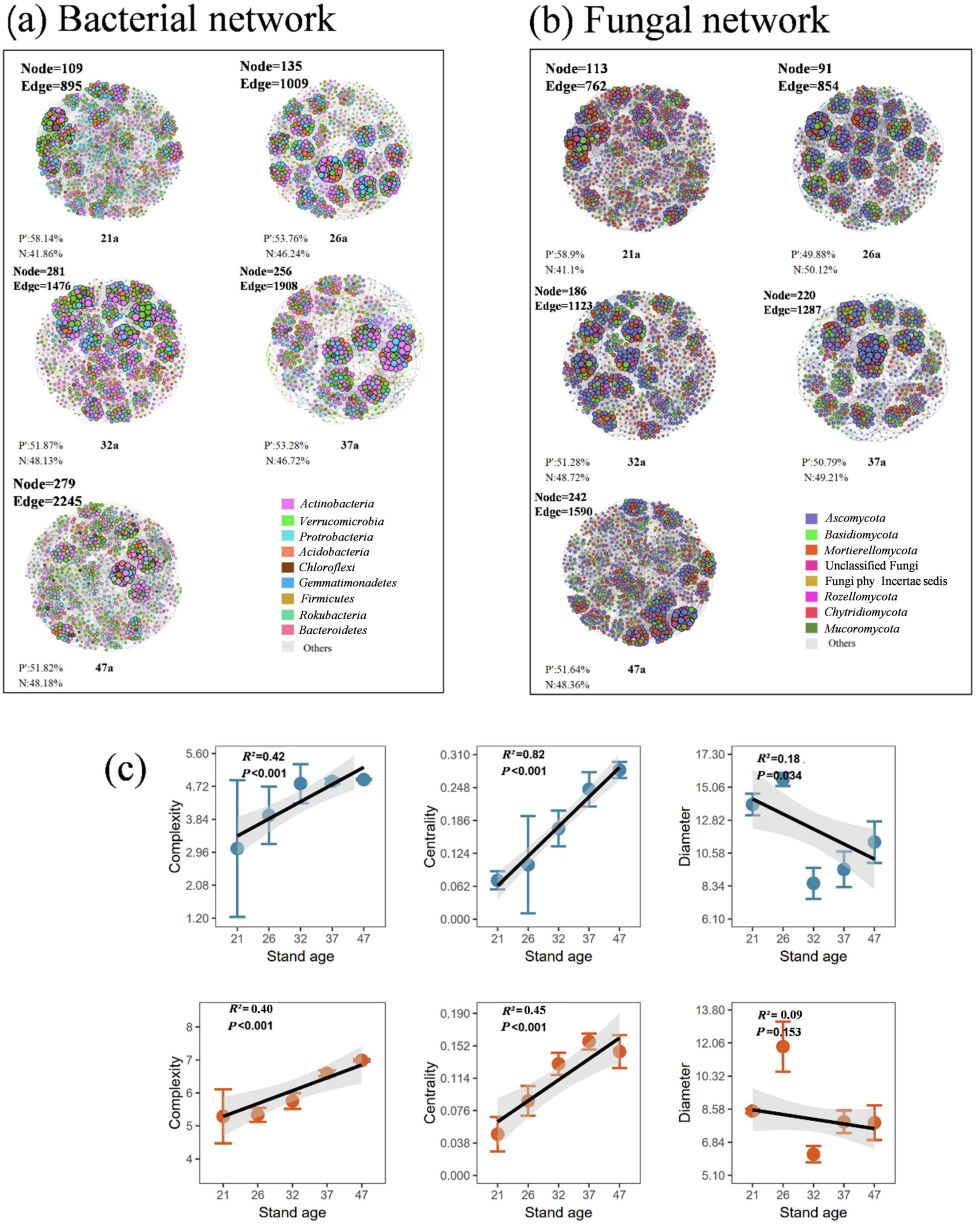
Co-occurrence network patterns of **(a)** soil bacterial and **(b)** fungal communities across different stand ages. Each node represents an amplicon sequence variant (ASV); node size reflects degree (number of connections), and node color denotes major phylum affiliation. P’ indicates a positive effect, whereas N denotes a negative effect. **(c)** Key topological properties of microbial co-occurrence networks, including network complexity, centrality, and diameter. Blue symbols indicate bacterial networks, whereas red symbols indicate fungal networks.

Both bacterial and fungal co-occurrence networks exhibited significantly stand age–dependent structural shifts (**Figures 4a, b**). For both bacterial and fungal co-occurrence networks, node and edge counts increased steadily with stand age, peaking in the 47a stands. Across all stands, positive correlations predominated over negative correlations in both bacterial and fungal networks, suggesting that cooperative or synergistic associations dominated microbial interactions.

Topological metrics further confirmed strong stand age effects on microbial network structure (**Figure 4c**). Network complexity increased significantly with stand age for both bacteria (R² = 0.42, *P* < 0.001) and fungi (R² = 0.40, *P* < 0.001). Network centrality also increased with stand age, showing a particularly strong relationship in bacterial networks (R² = 0.82, *P* < 0.001) and a moderate but significant increase in fungal networks (R² = 0.45, *P* < 0.001). In contrast, network diameter decreased significantly with stand age in bacterial communities (R² = 0.18, *P* < 0.05) but showed no significant trend in fungal networks (*P >*0.05).

### 3.4 Predictors of soil multifunctionality across multiple thresholds

**Figure 5:**
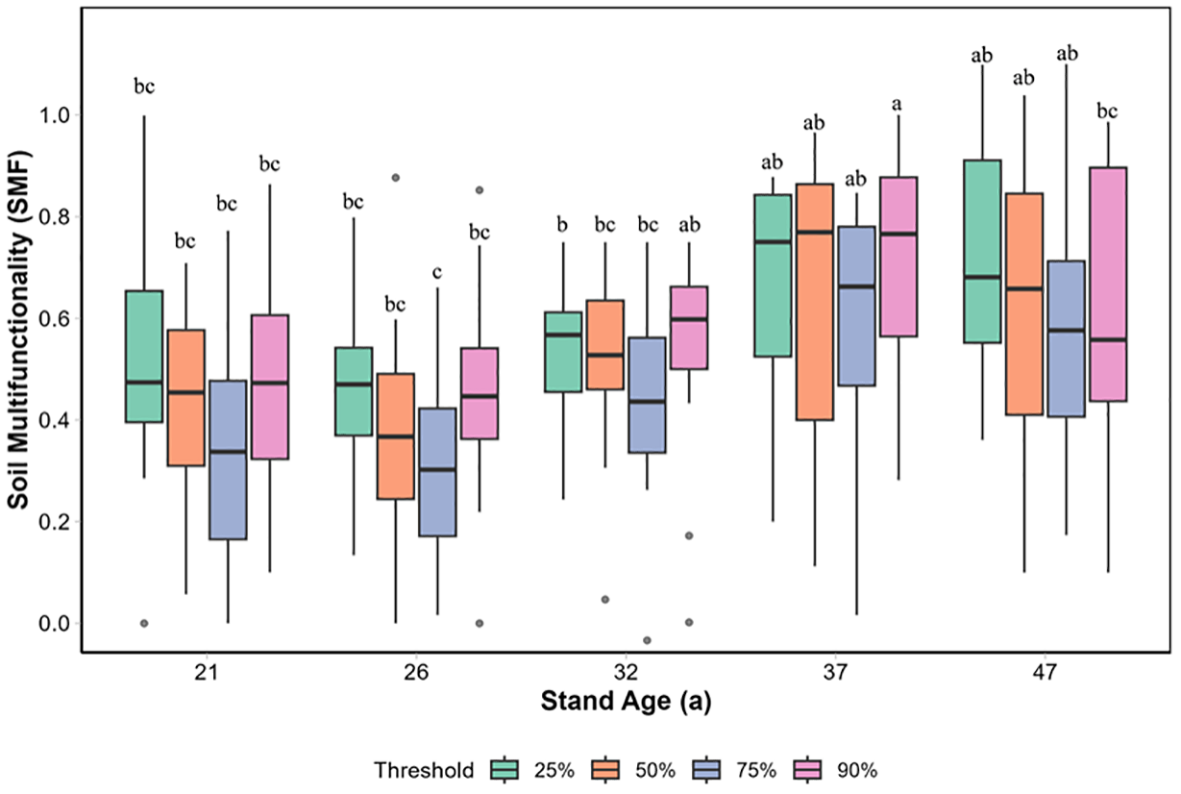
Soil multifunctionality (SMF) across different stand ages. SMF was evaluated at four thresholds: 25% (SMF_25), 50% (SMF_50), 75% (SMF_75), and 90% (SMF_90). Across all thresholds, SMF consistently peaked in mature stands. Different lowercase letters indicate significant differences (p< 0.05) among groups.

The variation of soil multifunctionality (SMF) across stand ages at four thresholds (25%, 50%, 75%, and 90%) is shown in **Figure 5**. SMF increased from young to mature stands and peaked at 37 years, with a slight decrease at 47 years across all thresholds.

**Figure 6:**
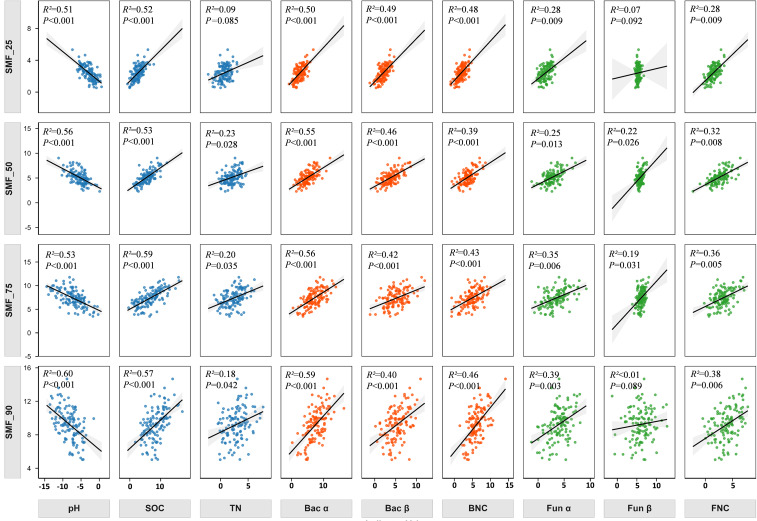
Linear regression model was used to illustrate the relationships between different microbial community attributes, soil chemical properties, and the soil multifunctionality (SMF) index. SMF was evaluated at four thresholds: 25% (SMF_25), 50% (SMF_50), 75% (SMF_75), and 90% (SMF_90). Microbial predictors included diversity and network complexity, while soil variables comprised soil organic carbon (SOC), total nitrogen (TN), and pH. Bac α, bacterial α-diversity; Bac β, bacterial β-diversity; BNC, bacterial network complexity; Fun α, fungal α-diversity; Fun β, fungal β-diversity; FNC, fungal network complexity.

SMF showed significant relationships with soil properties and microbial attributes across all multifunctionality thresholds (P< 0.05; **Figure 6**). Soil pH was negatively associated with SMF at all thresholds (R² = 0.51–0.60, *P* < 0.001), whereas SOC exhibited strong positive relationships with SMF, explaining a substantial proportion of SMF variation across thresholds (R² = 0.52–0.59; *P* < 0.001), A non-significant positive correlation was observed between TN and SMF (R² = 0.09–0.23; *P* = 0.028–0.085). Microbial community attributes also showed clear and threshold-consistent effects on SMF. Bacterial α-diversity and β-diversity were strongly and positively correlated with SMF across all thresholds (Bacterial α-diversity: R² = 0.50–0.59; Bacterial β-diversity: R²=0.40–0.49; all *P* < 0.001). In contrast, BNC was positively related to SMF, with its explanatory power increasing at higher thresholds (R² = 0.39–0.48, *P* < 0.001). Fungal attributes exhibited similar but generally weaker patterns. Fungal α-diversity showed consistent positive relationships with SMF across thresholds (R² = 0.25–0.39, *P* < 0.001), whereas fungal β-diversity explained limited variation (R²<0.01). FNC was positively associated with SMF, with stronger relationships observed at higher multifunctionality thresholds (R² = 0.28–0.38, *P* = 0.005–0.009).

### 3.5 Direct and indirect controls of soil multifunctionality

**Figure 7:**
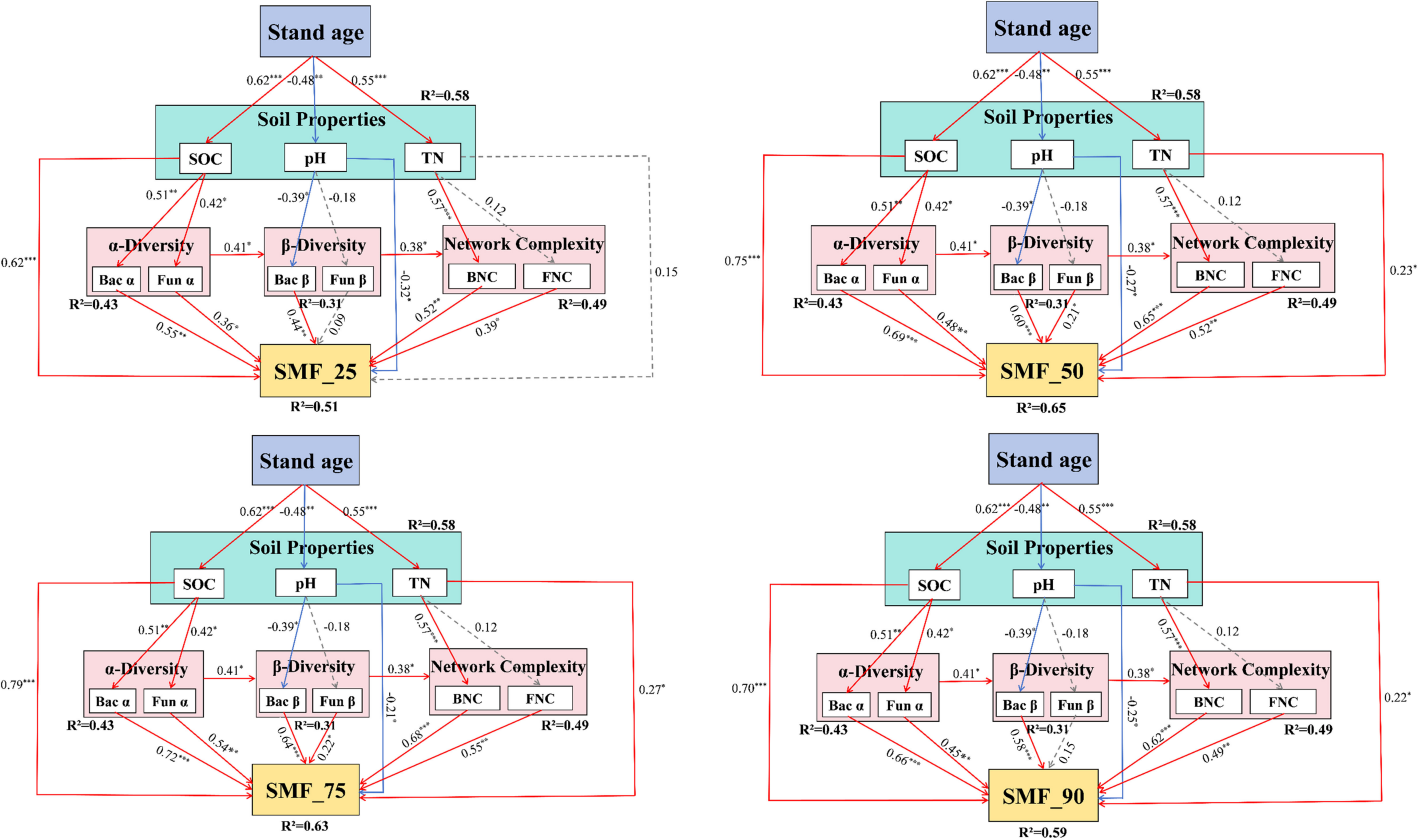
Path diagram of the structural equation model between soil multifunctionality (SMF) and driving factors. Panels (a-d) correspond to SMF at the 25%, 50%, 75%, and 90% thresholds (SMF_25, SMF_50, SMF_75, and SMF_90), respectively. SOC, the soil organic carbon; pH, soil pH; TN, total nitrogen; Bac α, bacterial α-diversity; Fun α, fungal α-diversity; Bac β, bacterial β-diversity (Principal Component 1); Fun β, fungal β-diversity (Principal Component 1); BNC, complexity of bacterial co-occurrence networks; FNC, complexity of fungal co-occurrence networks. For bacterial and fungal α-diversity, a composite index of Shannon and Chao1 was used (see Methods 2.8 for details). Solid red arrows indicate significant positive effects, solid blue arrows indicate significant negative effects, and dashed gray arrows denote non-significant relationships. The R² values represent the proportion of variance explained by each model. Model fit statistics:χ²=27.614, *P* = 0.319, AIC_C_=-116.783. *P < 0.05, **P < 0.01, ***P < 0.001.

**Figure 8:**
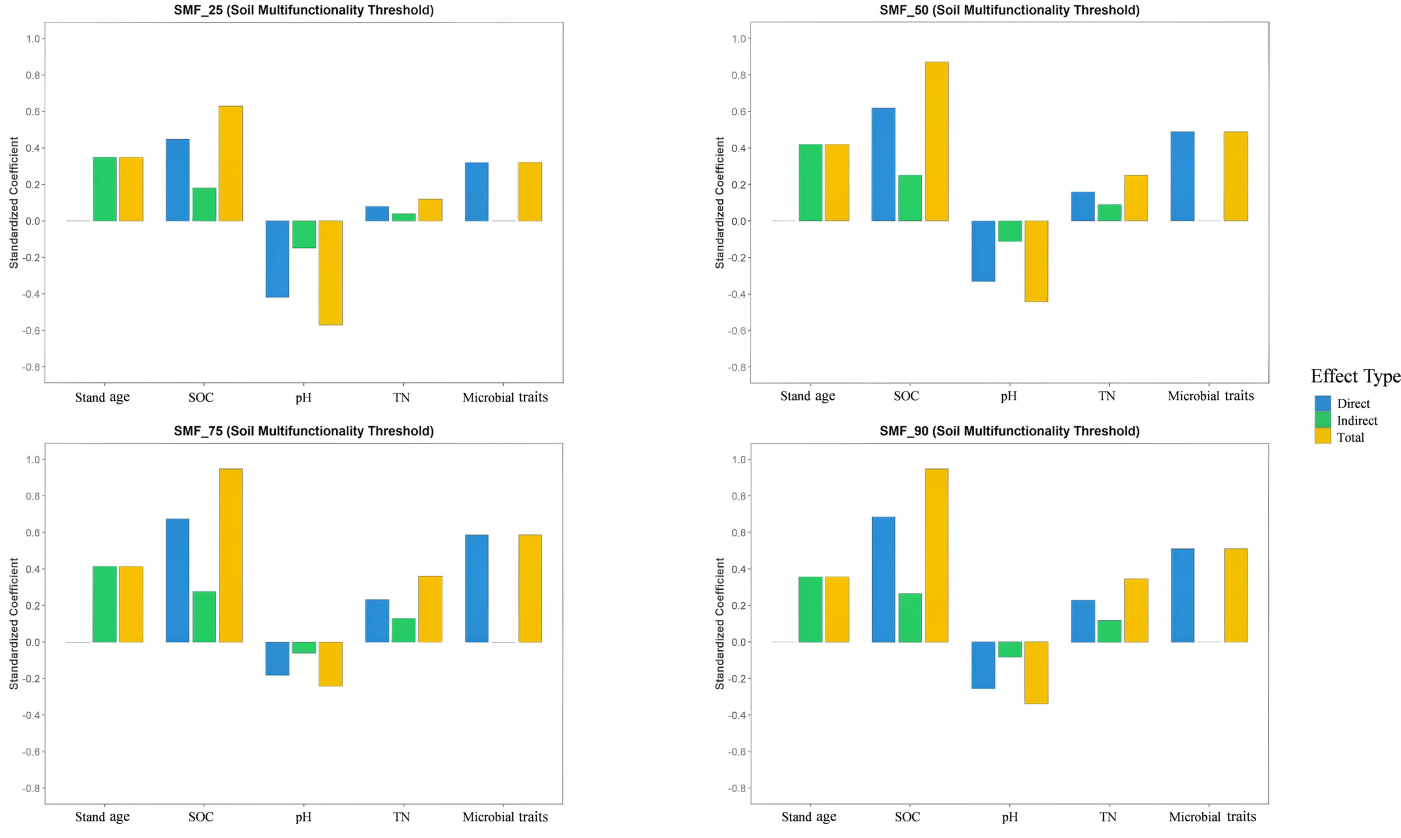
Standardized direct and indirect effects of abiotic and biotic predictors on soil multifunctionality across multiple thresholds. Panels (a-d) represent SMF at the 25%, 50%, 75%, and 90% thresholds (SMF_25, SMF_50, SMF_75, and SMF_90), respectively. Microbial traits include bacterial α-diversity (Bac α), fungal α-diversity (Fun α), bacterial β-diversity (Bac β), fungal β-diversity (Fun β), bacterial network complexity (BNC), and fungal network complexity (FNC). All other abbreviations are explained in **Figure 7**.

SEMs explained 51–68% of the variation in SMF across all threshold levels (**Figure 7**). Stand age influenced SMF predominantly through indirect pathways mediated by soil nutrients and microbial attributes (**Figure 8**). SOC was positively associated with SMF (*P* < 0.05) and also exerted indirect effects via increased microbial α-diversity. TN exhibited significant positive correlations with SMF at all thresholds except 25% (*P* < 0.05). Across all threshold levels, the contribution of TN to SMF was largely mediated by indirect pathways, primarily through increases in BNC, which in turn positively influenced SMF. In contrast, pH, which was also linked to stand age, consistently showed negative indirect effects on SMF (*P* < 0.05), largely through alterations in microbial β-diversity. Among microbial factors, microbial α-diversity, bacterial β-diversity, and network complexity (both BNC and FNC) were positively related to SMF across all threshold levels (*P* < 0.05). However, fungal β-diversity showed no significant effects on SMF at the 25% and 90% threshold levels (*P* > 0.05; **Figures 7a, d**).

## 4 Discussion

### 4.1 Variation in soil physicochemical properties along the stand age gradient

Our results demonstrated that stand development induces a systematic reorganization of soil physicochemical conditions that underpins variation in soil multifunctionality (SMF), consistent with previous findings (Ma et al., 2018; Liu et al., 2021). Along the chronosequence, most nutrient indicators (SOC, TN, TP, AP, and AK) followed a unimodal trajectory, increasing from early to mid-aged stages and declining in the mature stages, whereas soil pH decreased monotonically with stand age. This pattern reflects a dynamic balance among litter inputs, nutrient uptake, and microbially mediated turnover during plantation development (Miao et al., 2014; Zhao et al., 2015). In young stands, rapid tree growth imposes strong nutrient demand while litter inputs remain limited, which constrains soil nutrient accumulation. As stands transition into mid-aged stages, increased litterfall and fine-root turnover correspond with organic matter inputs, while declining growth rates reduce nutrient consumption, collectively promoting nutrient accumulation in surface soils. In contrast, the nutrient decline observed in 47a stands likely reflects long-term nutrient extraction combined with reduced nutrient-use efficiency under increasingly acidic conditions (Ma et al., 2018; Zhang et al., 2020). Persistent soil acidification, associated with organic acid inputs from coniferous litter and sustained microbial respiration, may further suppress nutrient mineralization and base cation availability, thereby reducing nutrient replenishment (Zhang and Sun, 2016; Gao et al., 2021). The pronounced vertical stratification of soil nutrients, with higher concentrations in the 0–10 cm layer, underscores the dominant role of surface litter decomposition and shallow root activity in regulating soil resource availability (Wang et al., 2012).

### 4.2 Variation in soil microbial diversity and community composition along the stand age gradient

Changes in soil microbial communities represent a central biological mechanism linking stand age to SMF (Adamo et al., 2021). Age-related increases in bacterial and fungal α-diversity were observed, with a subsequent decline in the 47a stand. This pattern supports the hypothesis that microbial diversity is maximized under intermediate resource availability and habitat heterogeneity but constrained under nutrient depletion and progressive acidification in older stands (Liu et al., 2022). The increase in stand age is typically accompanied by the accumulation of aboveground biomass, improvements in soil hydrothermal conditions, and elevated nutrient levels, which collectively provide a more favorable habitat for microorganisms. Simultaneously, changes in litter input, fine root biomass, and their decomposition rates accelerate nutrient cycling processes, thereby markedly shaping microbial community structure and diversity (Chen et al., 2018; Liu et al., 2020). At the mid-aged stages, declines in litter quality, increased complexity of chemical composition, and soil acidification may suppress or even eliminate susceptible microbial groups, leading to reduced diversity.

Bacterial communities exhibited stronger compositional differentiation across stand ages than fungal communities, consistent with their higher growth rates and greater sensitivity to short-term environmental variation (Gao et al., 2021). *Actinobacteria* and *Proteobacteria* play pivotal roles in carbon and nitrogen cycling (Větrovský et al., 2014; Delgado-Baquerizo et al., 2018), whereas *Ascomycota* and *Basidiomycota* occupy crucial ecological niches in the degradation of recalcitrant organic matter and litter turnover (Cairney, 2005; Paungfoo-Lonhienne et al., 2015). Although stand age significantly influenced community structure, the dominant bacterial and fungal phyla remained prevalent across all stand ages. This indicates that these taxa possess a broad ecological niche width and strong environmental adaptability (Yang et al., 2015; Kielak et al., 2017). Nevertheless, significant separation detected by PCoA indicates that stand age reshaped community composition primarily through shifts in subordinate taxa rather than overall species replacement.

### 4.3 Progressive enhancement of microbial network complexity with stand development

This study demonstrated that both bacterial and fungal networks exhibited a clear increase in complexity and connectivity with increasing stand age, consistent with observations from forest succession studies (He et al., 2022). This progressive enhancement of network complexity likely reflects improved resource availability, intensified niche differentiation, and increased metabolic interdependence among microbial taxa as stands mature.

Across all stand ages, microbial networks were dominated by positive correlations, indicating that cooperative or facilitative interactions prevail during stand development. Such synergistic interactions contribute to more tightly connected and efficient network structures, which are essential for maintaining soil biological stability and promoting nutrient cycling (Bashir et al., 2020). Notably, bacterial networks showed stronger and more consistent increases in connectivity than fungal networks, likely due to the higher functional redundancy and faster turnover rates of bacterial communities, which enhance their resilience to stage-specific resource competition. In contrast, the fungal network exhibited a transient reduction in node number at the early stage (26a), a pattern not observed in bacterial networks. This fluctuation may be attributed to the greater sensitivity of fungi, particularly mycorrhizal fungi, to shifts in substrate quality and nutrient stoichiometry during stand development. Such sensitivity may lead to temporary restructuring of fungal associations before stabilizing in later stages. In the 47a stand, network complexity reached its highest level, while the rate of decline in network diameter slowed and microbial diversity showed a slight decrease. This pattern suggests that microbial networks in mid-aged forests may be approaching a relatively stable saturation state, in which further increases in connectivity do not necessarily translate into proportional functional gains (Zhu et al., 2010). If nutrient inputs become limiting beyond this stage, negative feedbacks on microbial interactions and ecosystem functioning may emerge. Overall, the tightly connected microbial network observed in older stands underscores its critical role in sustaining soil processes and enhancing ecosystem stability during forest development.

### 4.4 Multi-pathway patterns of covariation with soil multifunctionality across stand ages

This study further confirmed that the formation and maintenance of SMF result from the coupled multi-pathway effects of biotic and abiotic factors (Doménech-Pascual et al., 2025). Soil SOC and TN exerted strong positive effects on SMF through both direct and indirect pathways, whereas soil pH imposed a persistent negative constraint across stand ages. Notably, these relationships exhibited clear threshold dependency, with stronger coupling effects observed at moderate multifunctionality levels. Overall, the positive contributions of SOC, TN, and microbial network complexity to SMF exceeded those of microbial diversity alone. Microbial network complexity, particularly bacterial network complexity, explained SMF variation more effectively than species diversity, especially at higher multifunctionality thresholds. This pattern strongly supports the emerging paradigm that interaction structure, rather than species richness per se, governs ecosystem multifunctionality (Yang et al., 2024). SOC primarily enhanced SMF by sustaining microbial α-diversity and providing a stable resource base, whereas TN influenced SMF mainly by strengthening bacterial co-occurrence network connectivity, highlighting distinct nutrient-regulation pathways.

Variations in soil chemical properties across the stand age chronosequence acted as the primary predictor for SMF development and maintenance. Progressive accumulation of SOC and TN with stand development, particularly in surface soils, created favorable conditions for the synergistic expression of multiple soil functions. This nutrient regime also acted as an environmental filter, promoting functionally advantageous microbial groups (e.g., *Actinobacteria*, *Proteobacteria*, and *Basidiomycota*) and reinforcing metabolic interdependence within microbial networks, thereby enhancing functional integration (Dang et al., 2017). In contrast, soil pH was linked to SMF primarily by constraining microbial turnover and β-diversity. Weakly acidic conditions favored oligotrophic taxa such as *Acidobacteria* and suppressed inefficient nutrient consumption, indirectly stabilizing soil functions despite the overall negative pH–SMF relationship (Jiang et al., 2024).

Differences between bacterial and fungal communities further revealed intrinsic heterogeneity in SMF-driving mechanisms. Owing to their faster turnover rates and higher environmental responsiveness, bacterial β-diversity and network connectivity closely tracked stand age succession and exerted consistent effects on SMF. In contrast, fungal communities characterized by longer life histories and greater functional conservatism contributed significantly to SMF only at intermediate functional levels (Zheng et al., 2025). The continuous enhancement of microbial network complexity thus reflects improved synergistic metabolism and resource allocation efficiency, providing critical support for SMF stability and resistance to disturbance (Zhai et al., 2024). Collectively, the pathway-specific coupling among soil nutrients, microbial diversity, and microbial network structure forms a composite driving system for SMF across stand ages (Liu et al., 2025). This multi-pathway synergistic framework appears robust across forest biomes and is fully applicable to larch plantations, underscoring its broad ecological generality.

Several limitations should be acknowledged. First, the SMF index was constructed using soil physicochemical properties and microbial attributes, without inclusion of independent functional process measurements such as enzyme activities or soil respiration rates. Following Delgado-Baquerizo et al. (2016), we acknowledge that our findings should be interpreted as correlative patterns rather than causal mechanisms. Because the SMF index shares component variables with several predictors, the reported associations partly reflect internal covariance within the dataset and should not be overinterpreted as independent predictive or causal effects. Second, the stand-age gradient in this study is based on a space-for-time substitution approach. While widely used in forest ecology, this design cannot replace long-term longitudinal monitoring. Thus, temporal extrapolation of the observed patterns should be made with caution. Third, our study was conducted in a single forest type (*L*. *principis-rupprechtii* plantations), which may limit the generalizability of the findings to other forest ecosystems with different species composition or environmental contexts. Finally, stand age integrates multiple structural attributes (e.g., tree height, DBH, canopy closure, and litter inputs) that were not explicitly quantified in this study but may jointly influence soil properties. Future work should incorporate these variables to better disentangle the direct effects of stand age from associated structural drivers.

## 5 Conclusions

This study clarified how stand age is linked to SMF in larch plantations by integrating soil nutrient dynamics, microbial diversity, and microbial co-occurrence network structure along a 47a chronosequence. SMF did not respond linearly to stand development but emerged from a coordinated, multi-pathway cascade linking abiotic resource availability with microbial community organization. Stand development systematically restructured soil physicochemical conditions, characterized by the accumulation and subsequent decline of SOC and TN, together with progressive soil acidification. These abiotic shifts acted as primary environmental filters shaping microbial community attributes. SOC corresponded with SMF mainly by promoting microbial α-diversity, whereas soil pH imposed a persistent negative constraint on SMF by regulating microbial turnover and β-diversity. Importantly, microbial co-occurrence network complexity, particularly within bacterial communities, explained SMF variation more effectively than microbial diversity alone, especially at higher multifunctionality thresholds. TN contributed to SMF predominantly through indirect pathways by strengthening bacterial network connectivity, highlighting that interaction structure and functional integration, rather than species richness per se, govern multifunctional soil processes during stand development. Collectively, these findings support an emerging ecological paradigm in which SMF is primarily controlled by the organization of microbial interactions under resource constraints. From a management perspective, the results underscore the need for stand age–specific strategies to sustain SMF in larch plantations, including enhancing organic matter inputs in early stages, maintaining microbial network integrity in mid-aged stages, and mitigating soil acidification in mature stages.

## Data Availability

The raw data supporting the conclusions of this article will be made available by the authors, without undue reservation.
