# Supplementary material for: Age-dependent predictors of soil multifunctionality in larch plantations: roles of nutrients and microbial networks
Source: Front Plant Sci. 2026 Jul 16;17:1844613. doi: 10.3389/fpls.2026.1844613 (PMC13419974; doi:10.3389/fpls.2026.1844613)
Supplement: Supplementary file 1 [file DataSheet1.pdf]

Supplementary Materials

1 Supplementary Figures and Tables

1.1 Tables

Supplementary Table 1. Principal component analysis of the soil nutrients.

Soil Variables	PC1	PC2
Variation explained(%)	71.3	15.2
TN	0.94	0.18
SOC	0.92	0.15
AN	0.89	0.22
AP	0.86	0.09
TP	0.83	0.20
pH	-0.78	0.35
SWC	0.42	-0.72
AK	0.38	-0.85
TK	-0.35	0.42

Note: SWC is soil water content; pH is soil acidity and alkalinity; SOC is soil organic carbon; TN is total nitrogen; TP is total phosphorus; TK is total potassium; AN is alkali-hydrolyzable nitrogen; AP is available phosphorus; AK is available potassium.

Supplementary Table 2. Principal component analysis of microbial community metrics.

Microbial Variables	PC1	PC2
Variation explained(%)	62.8	20.5
Bac α-diversity	0.91	0.25
Bac β-diversity	0.75	-0.42
BNC	0.88	0.18
Fun α-diversity	0.84	0.30
Fun β-diversity	0.68	-0.38
FNC	0.82	0.15
Bac other network metrics	0.45	0.12
Fun other network metrics	0.38	0.10

Note: Bac α-diversity, Bacterial α-diversity; Bac β-diversity, Bacterial β-diversity; BNC, Bacterial network complexity; Fun α-diversity, Fungal α-diversity; Fun β-diversity, Fungal β-diversity; FNC, Fungal network complexity; Other network metrics are composite indices from supplementary topological parameters (nodes, edges, diameter, clustering coefficient, modularity, density, and path length). Not selected for final modeling due to lower explanatory power.

Supplementary Table 3. Raw sequencing depth of each sample before rarefaction.

**SampleID**	**Raw_Sequencing_Depth(reads)**
Sample1	7800
Sample2	8508
Sample3	7852
Sample4	8628
Sample5	10262
Sample6	8402
Sample7	7532
Sample8	5517
Sample9	5300
Sample10	5369
Sample11	4732
Sample12	5035
Sample13	8727
Sample14	8414
Sample15	6270
